# Unpacking a female language teacher’s identity transformations: a perspective of multiple I-positions

**DOI:** 10.3389/fpsyg.2024.1291940

**Published:** 2024-02-09

**Authors:** Huanling Xing, Liyan Liu, Anne Li Jiang, Neil Hunt

**Affiliations:** ^1^Institute of Teacher Education, Northeast Normal University, Changchun, China; ^2^School of Foreign Languages, Northeast Normal University, Changchun, China; ^3^Centre for English and Additional Languages, Lingnan University, Tuen Mun, Hong Kong SAR, China

**Keywords:** teacher identity, identity transformation, dialogical self theory, narrative inquiry, Chinese female teacher

## Abstract

The narrative inquiry investigates the construction and evolution of a female Chinese language teacher’s identity across her pre-service and in-service phases. Utilising data from interviews, class observation and written reflections, the research examines how internal and external aspects shape her identity development. It specifically explores the role of third positions, meta positions, and promoter positions drawing on the dialogical self theory. The findings reaffirm that a teacher’s identity is fluid and influenced by personal and professional factors. Over time, however, strong teaching beliefs and a growth mindset emerge as pivotal drivers for sustained and positive teacher development. The paper concludes by offering implications for pre-service teacher education and female teachers’ continuing professional development.

## 1 Introduction

The comprehension of language teaching and learning is commonly acknowledged to necessitate an understanding of language teachers, especially concerning their professional and personal identities (Beijaard et al., 2004; Varghese et al., 2005; Barkhuizen, 2017; Jiang and Zhang, 2021). Teacher identity development involves the exploration of personal concepts, beliefs and values influenced by personal factors as well as professional factors (Beauchamp and Thomas, 2009; Reeves, 2009; Jiang, 2022). This process varies for every teacher since teachers possess dynamic characters and undergo unique socialization processes with diverse prior experiences (Pillen et al., 2013a; Pennington and Richards, 2016).

Previous research has established that identity development is a situational process influenced by multiple factors, such as cultural factors, organizational roles, gender, and so forth (Brewer et al., 2002; Hou et al., 2023a). To contribute to this body of knowledge, our study was specifically carried out in China, a country characterized by a high-power distance and a collectivist cultural tendency. Within the collectivist paradigm prevalent in China, individuals derive self-knowledge primarily from their interdependence with others within their social groups, prioritising the needs of the collective (Woodhams et al., 2014; Triandis, 2018; Hou et al., 2023b). This cultural backdrop creates distinct challenges for identity development among Chinese female teachers, as underscored by Gao et al. (2022), who note that challenges may arise from the emphasis on interdependence and the prioritization of collective needs over individual ones. Examples of potential challenges include navigating societal expectations, striking a balance between personal aspirations and group harmony, and effectively managing roles within various social circles (Zhu et al., 2020). Despite the significance of these challenges, there exists a notable gap in research that delves into the long-term drivers of female teachers’ identity development within the Chinese cultural context. This gap is particularly noteworthy given the substantial representation of female teachers in the workforce and the distinct barriers they encounter in professional development (Ruan and Zheng, 2019; Luo et al., 2021). Additionally, while existing research has explored unique identity formation trajectories for both pre-service and in-service teachers, there is limited examination of teacher identity development covering both pre- and in-service periods. Recognizing the differences in situational contexts, it is essential to illustrate the fuller changing process that occurs in these two distinct yet continuous periods.

To address the above limitations, this research employs dialogical self theory to gain insights into the process of identity transformation of a female Chinese language teacher spanning the pre-service and in-service periods. The findings reveal a longitudinal process of identity transformation, highlighting the active agency of these changes. Additionally, they provide valuable insights into how teacher education programs can support female teachers in achieving sustainable lifelong professional development.

## 2 Literature review

### 2.1 Teacher identity

Teacher identity has a strong impact on the decisions teachers make about their teaching practice, the teaching content they choose, the relationships they maintain with their students (Beijaard et al., 2004), as well as whether and how they seek out professional development opportunities (Hammerness et al., 2007). The process of teacher identity formation reflects how teachers understand themselves as a teacher, how they act accordingly, as well as how they grow and develop. There is a general agreement in four characteristics concerning teacher identity formation (Ye and Zhao, 2018). Firstly, identity is in a state of flux, unstable and changeable. Secondly, identity is framed by internal and psychological processes as well as political, economic, cultural, and historical factors, resulting from continuous negotiation between personal and contextual factors. Thirdly, teachers constantly construct or reconstruct the substance of their experience via stories. Finally, identities form and develop in connection with others and are accompanied by emotions (Clarke, 2008; Song, 2016). In other words, from a post-structuralist perspective, identity is seen as multiple, dynamic, and a site of contradiction and struggle (Norton, 2013).

To date, the predominant focus of studies has been on delving into the formation and progression of the identities of either pre-service or in-service teachers within specific educational settings. For instance, Kayi-Aydar (2015) provided insights into the identity (re) negotiations and agency of three pre-service teachers enrolled in a research university in the United States, uncovering the intricacies of identity development within the realm of higher education. In a parallel vein, Ruohotie-Lyhty (2013) conducted a narrative study, comparing the professional identity formation of two newly qualified teachers in Finnish suburban basic schools, shedding light on the unique challenges faced by novice educators in diverse educational environments. Wei’s (2021) narrative inquiry explored the evolving professional identities of an experienced Chinese teacher, probing into the intellectual-rational and social-political dimensions of professional identity. This study significantly contributes to understanding the complexities of identity transformation among seasoned educators in the Chinese educational landscape. Ye and Zhao’s (2018) investigation delved into how three secondary school teachers developed distinct teacher identity trajectories amid changes in China’s educational policies, providing nuanced insights into how external factors shape individual teacher identity paths.

The collective findings highlight that prior research predominantly focused on teacher identity development within a single career stage, be it pre-service or in-service. However, acknowledging the widely accepted notion that teachers are lifelong learners (Day, 1999), and recognizing the benefits derived from both formal learning in school and informal learning in the workplace (Hoekstra et al., 2009), it is necessary to explore the continuity of professional learning and unravel the long-term influencing factors spanning both pre-service and in-service periods.

Moreover, literature underscores the importance of addressing tensions within the professional development landscape. These tensions may arise from conflicting expectations, differing cultural norms, and evolving educational policies, thereby creating a complex backdrop for teacher identity formation (Pillen et al., 2013b; Robertson and Yazan, 2022). Additionally, considering the impact of gender on conflict coping (Holt and DeVore, 2005) and the distinctive barriers faced by Asian female professionals (Woodhams et al., 2014), it becomes imperative to incorporate these nuanced factors in the exploration of female teacher identity development.

The objective of the present study is to illustrate the evolution of a female teacher’s identity, spanning both pre-service and in-service phases. The research seeks to understand the factors that contribute to the ongoing professional development of teachers by visualizing the continuous interaction of a variety of I-positions, as derived from dialogical self theory.

### 2.2 Theoretical framework: dialogical self theory

**Figure 1:**
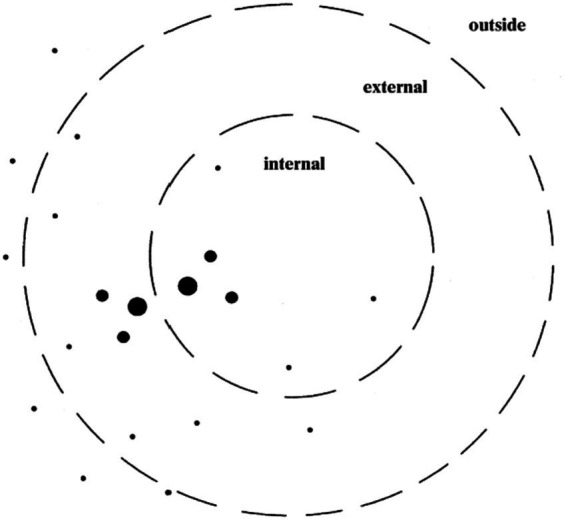
Positions in a multi-voiced self (Hermans, 2001a).

Dialogical self theory (hereafter, DST) is a psychological framework that weaves two notions, self and dialogue, inspired by both James (2012) classic notion of “the extension of the self” and Bakhtin’s (1981) “polyphonic novel.” DST conceptualizes the self as a spatial site in which a dynamic multiplicity of I-positions evolves over time and space, as can be visualized by the dots arranged in two concentric circles in Figure 1. Each position is characterized by one or more voices, contributing to a polyphonic nature of dialogism. This polyphony has the potential to generate new positions, establishing a dual relationship between position and voice. Positions can vary in the strength of their voices, and the interaction among voices can give rise to the creation of novel positions, each accompanied by a distinctive voice (Hermans and Dimaggio, 2007; Monereo and Hermans, 2023).

According to DST, an I-position refers to a specific voice that can be conceptualized as a narrative stance within a multifaceted self (Hermans, 1996; Hermans, 2001a). There are two main types of I-positions: internal positions and external positions. Internal positions exist within the inner domain of a person, such as I-as-a-mother or I-as-a-passionate-person. In contrast, external positions are situated outside of the person, involving relationships with entities like one’s child, mentor, or students (Hermans, 2001b; Grimell, 2018). The external and internal I-positions collectively constitute personal position repertoires (Kluger et al., 2008; Monereo and Hermans, 2023). The I-positions within personal position repertoires are further categorized into four types according to their functions: core position, third (mediator) position, promoter position, and meta position. A core position stands out due to its centrality, exerting influence over a substantial number of other positions within its domain (Raggatt, 2007; Leijen and Kullasepp, 2013). A third position serves as a mediator, emerging to alleviate conflicts that arise between two or more initial positions (Monereo and Hermans, 2023). A promoter position adds value to the entire array of positions, functioning as a guiding compass for the development of the position repertoire. A promoter position may include significant others or inspiring figures (Hermans and Gieser, 2011; Meijers and Hermans, 2018). Lastly, a meta position operates as an observer, enabling the self to transcend itself and adopt a “helicopter view” (Raggatt, 2007). Its function involves gaining a comprehensive understanding of the organization and interaction among various positions.

The rationale for applying DST as the theoretical framework for this study is grounded in the understanding that teachers’ professional identities are not static but rather exhibit both continuous and discontinuous developments throughout their teaching careers. This dynamic process occurs as teachers continually (re) position themselves, both internally and externally, in relation to various frames of reference, including their colleagues, their students and their families (Bullough and Draper, 2004). The personal position repertoires helped to gain insight into the organization of the self and the dialogue that takes place within the I-positions. The functioning of different positions is pinpointed to show how the position changes facilitate teacher identity development.

## 3 Methodology

Narrative inquiry develops with a precondition that people experience and live their lives in the form of stories, relating to people and events through the continuous construction of plots that have beginnings, middle, and end points (Sarbin, 1986; Clandinin and Connelly, 2004). We adopted this approach in our study because, as widely used in teacher identity research, it allows researchers to capture the complex and dynamic process of identity formation through the stories teachers live by (Akkerman and Meijer, 2011).

### 3.1 Research context and the participant

In 2007, the Chinese government launched the policy of “Government-funded Normal Students” (GFSs), which was a major initiative undertaken to address the educational inequalities that existed in less advanced areas or schools (The State Council, 2007). The policy aimed to attract and support talented students from rural areas or low-income families to pursue a career in teaching and equip them with the necessary skills and knowledge to effectively serve the communities where they came from, most of which are less advanced areas or rural areas. To implement the policy, the government selected six top normal universities in China as pilot sites to provide four-year pre-service teacher education for the GFSs.

As a product of this policy, GFSs are distinctive in some important ways. Once enrolled, they can enjoy a series of preferential treatments. They will be provided with a monthly allowance and free tuition and accommodation fees. However, they must return to their home provinces and work in the educational system as teachers for a minimum of 6 years after graduation. They can either seek teaching positions in their provinces by themselves or depend on the local education bureau to assign one to them.

Ying, the participant in this research, is a GFS who graduated in 2011 and returned to her hometown. Her teaching position was assigned by the local education bureau. Ying mentioned in her reflective journal that most teachers she worked with focused on drilling exercises, and the classes were teacher-centred while most of the students she taught lacked the motivation to study and did not have clear plans for their future. The English language level of most students was also below average compared to other local schools.

Ying is a university classmate and a long-time close friend of the first author. They have kept constant communication to share their teaching experiences as well as the changes including critical events, complaints, and disappointments in life and career, which is an advantage for this research since identity formation involves not only stories with people and events in the profession but also changes of personal belief over time and critical events happening in life (Leeferink et al., 2018).

### 3.2 Data collection and analysis

This study’s narratives evolve from a long-term connection between Ying and the first author, originating from their shared experiences as preservice teachers and extending to the composition of this paper in 2022. Multiple qualitative data were collected over the past 12 years, including reflective journals, classroom observations, and interviews. Initially, the narrative was developed through Ying’s reflective journals, written over the 12 years for personal reflection. It was later supplemented by the first author’s observations of Ying’s classes, specifically focusing on her internal and external positions in classes. These narratives underwent further refinement as the first author responded to reflective journals and classroom observations by sharing her own experiences and seeking more information. Additionally, three semi-structured interviews conducted in 2022 on the campus where they spent their college years played a key role. These interviews, each lasting about two hours, served as crucial junctures for examining personal position repertoires and reflecting on the development and functioning of I-positions during different transitional stages in Ying’s 12 years of teaching and learning.

The authors engaged in a systematic and iterative analysis of the collected materials over the course of the data collection process. Ying’s narratives were transcribed verbatim, and the analytical procedures unfolded as follows: (1) Sorting and labeling: this initial phase involved the chronological organization of all collected materials. The materials were thoughtfully categorized into three distinct stages, providing a foundational structure for subsequent analysis. (2) Coding and categorizing: in the second phase, a detailed coding process was implemented. Noteworthy I-positions were identified within their respective temporal contexts, and these were then systematically classified into categories. This step ensured a nuanced understanding of the evolving narrative landscape. (3) Data analysis: the final phase of data analysis was conducted within the framework of dialogical self theory. The focus was on the identification and negotiability of functions embedded in the I-positions. Additionally, a keen emphasis was placed on unraveling the intricate interactions among I-positions, particularly during transitional phases across various stages.

Throughout the analytical process, the narratives were revisited to unravel the participant’s underlying intentions and motives, influenced by beliefs and values possibly unknown to the participant herself. In a commitment to participant feedback, Ying was invited to comment on the final draft of the data analysis, providing valuable insights that significantly contributed to the refinement of the final analysis. This participatory approach enhances the trustworthiness of the research findings.

## 4 Findings

This section presents the interplay of internal and external positions in Ying’s teaching profession during different periods of her career. The characteristics of the interplay mark three identity transformations interconnected with events, people and objects functioning as third positions, promoter positions and meta positions.

### 4.1 Transition 1: harmonious chorus of internal and external voices

**Figure 2:**
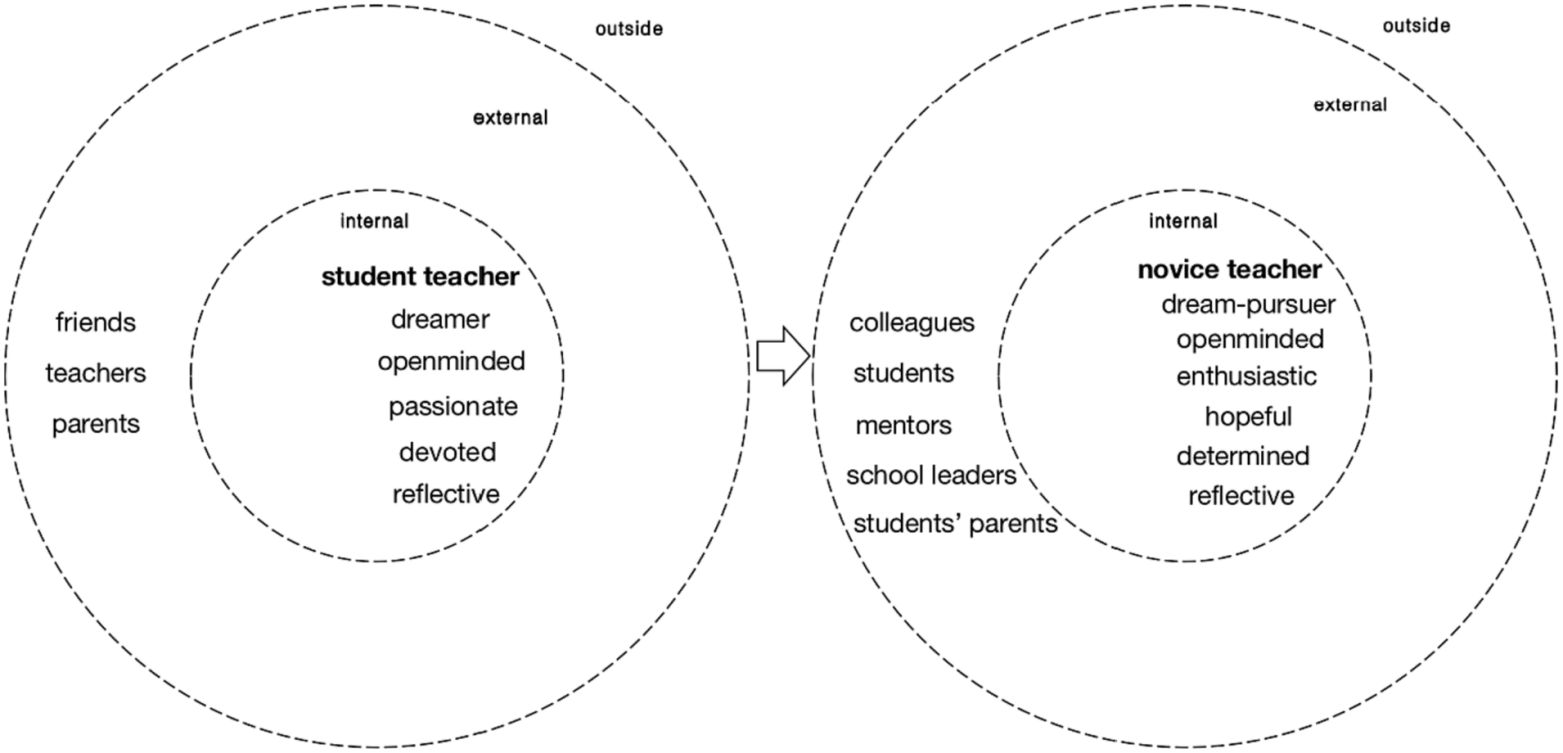
Personal position repertoire of Ying as a student teacher (left); personal position repertoire of Ying as a novice teacher (right).

Ying’s internal core position as a student teacher gives way to her I-position as a novice teacher after she graduated from university and started working in junior high school (see Figure 2). Being a student teacher used to be Ying’s core position, around which some sub-positions like as-a-positive-person and as-a-devoted-person existed, and she was devoted and passionate about being a teacher. She was an open-minded person since she was eager to learn from her teachers. The teachers she modelled after showed the same characteristics, as she said:

When I was a student, I was determined to be a teacher in the future. I had good relationships with my teachers and modelled after them, especially those who were willing to accept new things and take challenges.

(5/2022, 1st round of interview).

The external positions such as her teachers had a strong influence on the development of Ying’s identity.

After Ying started teaching in a junior high school, her core position changed to a novice teacher. Correspondingly, some new external positions emerged, like her colleagues, her students, and her mentors and a multiplicity of voices appeared. Despite that, different I-positions stayed in harmony and no acute contradiction emerged. Ying was still quite enthusiastic about her teaching and hopeful about the future. These internal and external positions function as promoter position clusters to keep the continuity of Ying’s teacher identity transformation, as Ying commented:

When I became a teacher, I devoted all my heart to teaching. I always prepared classes or tutored students wholeheartedly. Other people’s recognition was important to me. If my colleagues and students said I was doing good or they learned a lot from me or my class, I would be happy the whole day…

(5/2022, 1st round of interview).

Although contradictions existed in this period, the internal positions, like as-a-reflective-person and external positions, such as the mentor, experienced colleagues, served as promoter positions to guide Ying in a positive way. As Ying commented:

It is not all smooth. For example, when I tried to be friends with the students, I found it hard to be strict with them. My colleagues and my mentor constantly reminded me to keep a proper distance from the students. I found it difficult to balance my thinking and real situations, but I was willing to take the challenges and see them as chances to learn.

(5/2022, 1st round of interview).

It appears that Ying enjoyed her position as a teacher and dedicated herself to the teaching practice the same as she was a student teacher. Although she was also faced with the challenges posed by external positions, like her students, she took a positive attitude and treated the challenges as chances to develop, which marked her first transition with fewer psychological difficulties (Helms-Lorenz et al., 2012). In other words, the external and internal positions do not contradict much so the continuity of teacher identity is guaranteed.

### 4.2 Transition 2: dominance of the external positions and powerless internal positions

Ying got married after two years of work and soon had a son. After Ying built her family, it was noticeable that the I-positions related to family outweighed positions in the teaching profession in number as well as importance. Ying’s position as a mother and wife in the family domain opposed and contradicted her position as a teacher in the professional domain. Specifically, constant topics in our conversations, as well as in Ying’s journals, revolved around the school’s continually evolving requirements, her colleagues’ complaints about her perceived lack of devotion, and the challenges arising from parental responsibilities and conflicts related to child-rearing, which nearly overwhelmed her.

Ying was struggling to balance her family duties and teaching career, gradually losing her enthusiasm and dream. Thus, the voice of Ying’s internal positions lost agency. “That was my dark age” as Ying put it in her diary. Meanwhile, the external positions dominated the whole dialogue in identity formation since she was constantly contradicted or challenged by her family and her school, as the following comment reveals:

My mother-in-law and my mother thought I should be the one taking care of the child or doing family chores. My sacrifice was duties that I could not complain about. My husband was busy with his career and not helpful, making me quite helpless and disappointed. I was quite anxious about my career. And I would also feel guilty if I didn’t spend more time with my child. However, my school, my colleagues and my students were also dissatisfied since I couldn’t devote my time and energy as usual. I felt guilty about being an incompetent mum, and an inefficient teacher…

(5/2022, 1st round of interview).

Amid this transition, marked by contradiction and disequilibrium, Ying experienced a sense of being lost and fatigued. In response to these conflicts, Ying progressively embraced a pragmatic approach, giving rise to the creation and evolution of internal third positions, such as that of an open-minded and inefficient mother. As she wrote in her diary:

I know I am not a perfect mum. I have not done all things well. If I can allocate time better, I will be more efficient. But I still need time to learn as a mother. Stay hungry, stay foolish.

**Figure 3:**
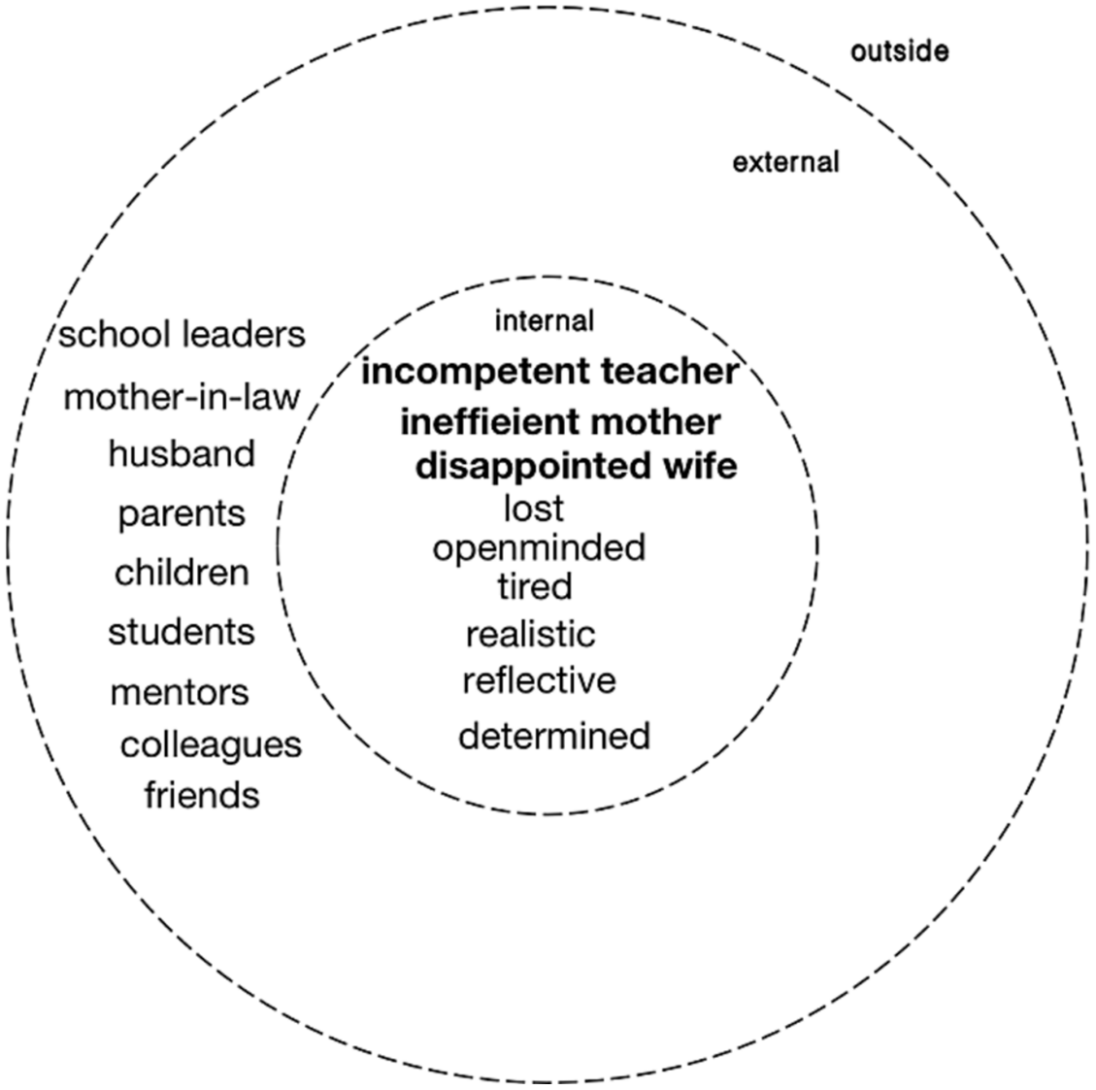
Personal position repertoire of Ying as a four-year teacher.

Simultaneously, external positions in both the professional and family domains wielded authoritative discourse, contrasting with internal positions characterized by feeble voices (Hermans, 2012; see Figure 3). In the professional realm, which included leaders, mentors, colleagues, and students, dissatisfaction with her work and impatience with her performance surfaced. Similarly, within the family domain, her mother-in-law interfered in her decisions and judged her choices. Faced with these challenges, her husband and mother assumed third positions, providing not only assistance and empathy but also actively sharing responsibilities as cooperative partners.

While she remained determined to be a teacher, the ability to balance multiple positions waned. The dilemmas faced by female teachers were evident in the dominance of external positions and the powerlessness of internal positions. However, positive third positions also emerged. The role of the third position was crucial in mediating conflicts and nurturing a harmonious self-dialogue within the framework of dialogical self theory.

### 4.3 Transition 3: diverse but stable internal positions and limited influence of the external positions

Ying’s identity undergoes a continuous evolution shaped by the influence of various I-positions, despite the complexity of the situation. Amidst this, a stable internal position remains – that Ying is an open-minded individual who embraces a willingness to learn.

Reviewing the longitudinal data reveals that when confronted with challenges, Ying consistently sought solace and solutions in conversations with her friends and family members, like her mother-in-law, as she stated:

My mother-in-law used to be an excellent primary school teacher. She told me a good teacher must be a good mother, which I didn’t agree with, but now I think it makes sense to some degree. Being a good mum and a good teacher requires the same thing that to know what children need and be there to help them. Because of her, I constantly plunged into reflection on being a mum and teacher.

(7/2022, 2nd round of interview).

Ying consistently shared her learnings from others, showcasing a commitment to knowledge exchange. Even in moments of great pressure, Ying demonstrated unwavering resilience, always determined to find a solution, as she commented:

When I was confused and tired, I observed how my female colleagues worked. I recalled those excellent teachers that I modelled when I was a student. I drew inspiration from them. As I raised my child, I got a different perspective to reflect on my relationship with my students. It dawned on me that they were children. The requirements I gave them were difficult like you must not sleep in classes, or you could not lose your temper… But they are children. Children need time to grow up, and mistakes are unavoidable. It’s the teachers’ and parents’ responsibility to be patient and guide them to grow.

(8/2022, 3rd round of interview).

Ying commenced a practice of attending to the diverse voices emanating from multiple I-positions. She sought counsel from her family, colleagues, and revered icons, displaying the functional role of the promoter position. This position operated as a guiding force, serving both as a model and a wellspring of inspiration, playing a pivotal role in molding Ying’s approach to both teaching and parenting. Concurrently, as Ying engaged in observations, discussions as well as reflection on teacher models and teacher-student relationships, a meta position came into play. This meta position facilitated a more expansive, cross-situational overview of Ying’s roles as a teacher and parent, fostering valuable insights into the interconnectedness of these positions. The functionality of the meta position instigated the ongoing formation and transformation of new perspective and insights. These new insights could be considered as novel third positions. These emerging third positions played a crucial role in stabilizing the organization of Ying’s self, providing adaptive responses to the evolving dynamics.

**Figure 4:**
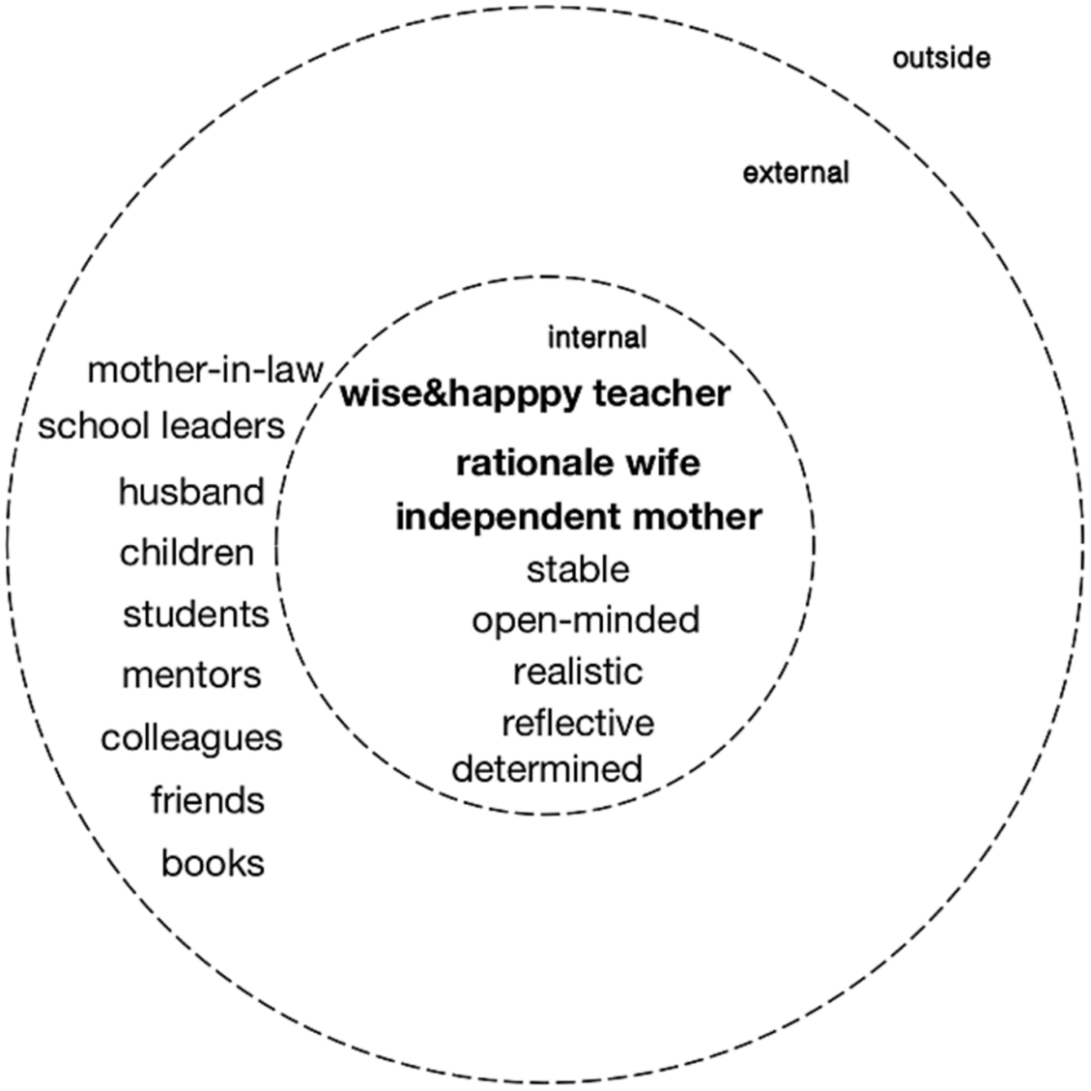
Personal position repertoire of Ying as an experienced teacher (12 years).

In conclusion, Ying’s I-positions as a teacher, mother, and wife coexisted in a complementary manner, illustrating a seamless integration facilitated by the collaborative interplay of promoter, meta, and third positions (see Figure 4).

## 5 Discussion

The study offers insights into the dynamic nature of teacher identity. Ying’s identity development involved reconciling various internal and external positions to create a coherent and continuous sense of self. The functioning of third positions, meta positions and promoter positions are illustrated and the dialogical space was explored (Akkerman and Meijer, 2011).

### 5.1 Continuity and discontinuity of a female teacher’s identity transformations: coalition of social and personal positions

Ying’s experiences are organized into a system of narrative structure and are re-storied in a way that illustrates her long-term personal and social position interaction which visualises her continuing professional development. All the narratives surrounding the “I” formed a developmental trajectory that showed how Ying grew from a student teacher into an experienced, happy teacher. Ying built her identity through the formation of different I-positions by telling narrative stories. In these stories, internal and external positions might change but always surround “I” as Hermans (2001a) asserted in line with James (2012). For example, there is a continuity between “the experiences of my child or my students” because all of them are extensions of the same self. In line with Bakhtin (1981), however, there is a discontinuity between the same characters as far as they represent different and perhaps opposing voices in the spatial realm of the self. For example, school leaders may think Ying is a good teacher because she devotes herself to teaching and responds to students’ needs in time; however, in the view of family members, Ying is an inefficient mother and teacher since she cannot balance life and work well. Discontinuity commonly exists in teachers’ careers, which can either bring tensions and burdens or facilitate development (Zwart et al., 2014).

### 5.2 Dynamic functioning of third position, promoter position and meta position

In the processes of identity transformations marked by both continuity and discontinuity, promoter positions, third positions, and meta positions assume distinct roles. Promoter positions affirm a consistency between prior and emerging identities. Despite existing research highlighting the challenges and burnout experienced by new teachers (Korthagen, 2016), Ying, in her initial transition as a novice teacher, maintained identity continuity, as evidenced by position interactions, and derived satisfaction from teaching and learning. This persistence can be attributed to her steadfast beliefs in teaching, particularly her resolute commitment and determination.

Drawing on DST, conflicts between two positions give rise to the emergence of a third position, which can reconcile and alleviate contradictions (Zhu and Chen, 2022; Monereo and Hermans, 2023). Ying encountered her most challenging period during marriage and childbirth when conflicting I-positions voiced simultaneously. However, the continuous emergence of third positions functioned as countervailing positions to maintain equilibrium among these conflicting voices, providing an opportunity for the formation of meta positions.

Meta positions play a pivotal role by providing a comprehensive overview across situations and serving a reflective function within the realm of I-positions (Hermans and Hermans-Konopka, 2010; Monereo and Hermans, 2023). In the diverse stages of her professional identity development, Ying demonstrated the adoption of various meta positions and reflective orientations. This aligns with established research that illustrates teachers’ diverse reflective orientations throughout different phases of their professional identity formation (Korthagen, 2016).

### 5.3 Beliefs and growth mindset: impetus for continuous identity transformation

Strong teaching beliefs and a growth mindset as the driving force for identity transformation are manifested in Ying’s narrative stories. As shown in Ying’s narratives, a dialogical space was formed along Ying’s identity transformation (Hermans, 2003; Uştuk and Yazan, 2023). This space was made up of several stable internal and external positions, which bore the characteristics of strong teaching beliefs and a growth mindset.

Strong teaching beliefs about how to be a good teacher ran throughout the whole teaching career. Ying was determined to become a good teacher since she was a student teacher and never wavered. The people she modelled after or the critical decisions she made never strayed away from her position as to-be-a-good-teacher. Teaching beliefs as an important part of teacher cognition have been proved to be significant in teacher’s professional development (Borg, 2003; Borg, 2018). This study reaffirmed its importance and visualised its mechanism of facilitating identity transformations.

**Figure 5:**
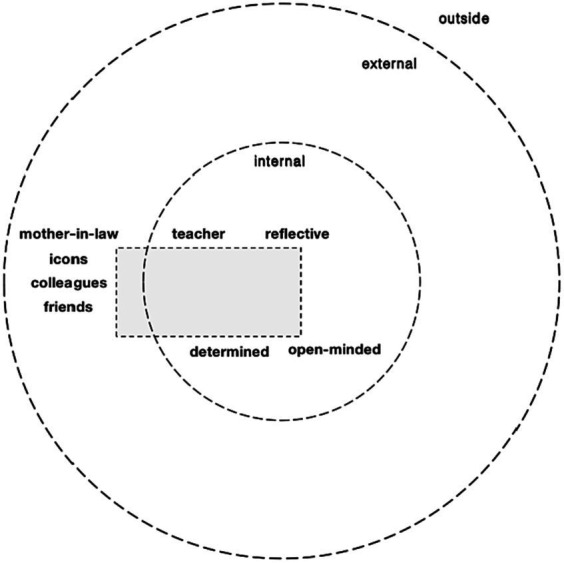
Main positions in Ying’s repertoire and the dialogical space.

Apart from the strong teaching beliefs, Ying showed distinctive characteristics of a growth mindset (Dweck, 2006; Yeager and Dweck, 2012). Hence, she was constantly reflecting on her being a teacher with references to other positions and stayed open-minded about any changes. As shown in her description, the icons and the colleagues she modelled after showed the same characteristics. Thus, when she was in a state of chaos, she would dialogue with them and practice their ways, which provided positive momentum for her development. Her mother-in-law and her friends played similar roles and gave stable voices in this dialogical space (see Figure 5). These internal and external positions together forming a new coalition, made a dialogical space and responded to the positions emerging or being overly dominant along the process (Hermans, 2003).

## 6 Conclusion and implication

Ying’s life narratives and the unfolding dynamics of her I-positions throughout her teaching journey provide valuable insights into the professional growth of Chinese female teachers. Our study underscores the effectiveness of dialogical self theory as a valuable tool for illustrating the continuous development of teacher identity (Zhu and Chen, 2022). This longitudinal research delves into the construction of a teacher’s identity from the initial teacher education program, offering fresh perspectives on teacher identity as an ongoing process of becoming.

Our findings suggest several implications for future research and teacher education. Firstly, the interplay of Ying’s I-positions reveals both continuity and discontinuity in her identity development, shedding light on unique pressures and barriers faced by Chinese female teachers. This includes societal expectations about women’s roles and the dual pressures from professional and family obligations experienced by Chinese female professionals (Triandis, 2018; Hou et al., 2023b). To address these challenges, we recommend that female teachers adopt a positive attitude toward achieving work-life balance, seek support from friends and role models, and remain open when facing challenges or high levels of pressure. This proactive approach can contribute to their overall well-being and success in both professional and personal spheres. Additionally, we advocate for increased support from policymakers, schools, spouses, and other family members. This support should encompass concrete measures such as flexible working arrangements, mentorship programs, and awareness campaigns to foster an understanding of the unique challenges faced by female teachers. Moreover, to facilitate a more balanced and fulfilling work-life integration, we propose that professional development programs be designed with a keen understanding of the unique developmental characteristics and needs of female teachers. This involves not only acknowledging their professional aspirations but also considering their personal and familial responsibilities. Such programs can contribute to a more supportive and inclusive educational environment.

Secondly, our nuanced exploration of the functioning of third positions, promoter positions, and meta positions visualizes the transformative process of teacher identity. The study emphasizes the significance of strong teaching beliefs and a growth mindset in identity transformations. In Ying’s case, we find that determined and stable teaching beliefs, coupled with a growth mindset, significantly influence identity transformations in the long run, benefiting teachers’ ongoing professional development. Thus, teacher education curriculums should incorporate the cultivation of student teachers’ teaching beliefs and their growth mindset. The inclusion of growth mindset interventions in curriculum design (Yeager et al., 2019) can be particularly impactful. This approach involves integrating activities that foster resilience, adaptability, and a proactive attitude toward professional growth. In essence, our study not only sheds light on the unique challenges faced by Chinese female teachers but also provides concrete recommendations to empower them in their professional journeys. Through a comprehensive understanding of the interplay between personal and professional dimensions, schools, educators and policymakers can work collaboratively to create a supportive and conducive environment for female teachers in achieving continual lifelong professional development.

While this study provides valuable insights into the dynamic process of a female teacher’s identity development, it is important to acknowledge certain limitations. The research predominantly focuses on the cultural and linguistic context of a high-power distance and a collectivist culture tendency, which may impact the generalizability of findings. To address this limitation, future research could incorporate diverse cultural perspectives. Additionally, our reliance on qualitative methods suggests the potential benefits of a mixed-methods approach to triangulate findings, introducing quantitative measures to complement our qualitative insights. Furthermore, the study primarily explores the teacher’s perspective, and future studies could enhance comprehensiveness by including the insights of teacher educators. We welcome ongoing research and hope this longitudinal study lays the groundwork for further exploration of teacher identity.

## Data availability statement

The original contributions presented in the study are included in the article/supplementary material, further inquiries can be directed to the corresponding author.

## Author contributions

HX: Conceptualization, Data curation, Formal analysis, Writing – original draft. LL: Conceptualization, Formal analysis, Writing – review & editing. AJ: Supervision, Validation, Writing – review & editing. HN: Supervision, Validation, Writing – review & editing.
